# 

**Published:** 1944

**Authors:** 


### The Bristol Medico-Chirurgical Journal

A JOURNAL OF THE MEDICAL SCIENCES FOR THE WEST OF ENGLAND AND SOUTH WALES

PUBLISHED UNDER THE AUSPICES OF

*THE BRISTOL MEDICO-CHIRURGICAL SOCIETY*

EDITOR

J. A. NIXON, C.M.G., M.D., F.R.C.P.

WITH WHOM ARE ASSOCIATED

A. E. ILES, O.B.E., M.B., B.S., F.R.C.S.

A. RENDLE SHORT, M.D., B.S., B.Sc., F.R.C.S.

W. MELVILLE CAPPER, M.B., B.S., F.R.C.S.

E. WATSON-WILLIAMS, M.C., Ch.M., F.R.C.S., Assistant Editor

"Scire est nescire, nisi id me Scire alius sciret"

ARMY MEDICAL APR 23 1946 LIBRARY

*VOL. LXI*

1944

BRISTOL: J. W. ARROWSMITH LTD.

W1
BR323

### Contents of Vol. LXI.

*Page*

The Thirty-Second Long Fox Memorial Lecture: The Social Aspects of Acute Rheumatism. By PROFESSOR C. BRUCE PERRY, M.D., F.R.C.P. .. .. 1

"Look and See," Foreign Bodies in Air and Food Passages. By E. WATSON-WILLIAMS, M.C., CH.M., F.R.C.S. .. .. .. .. .. .. 11

Edward Tyson, M.D., F.R.S., 1650–1708 .. .. .. .. .. .. 15

The Goodenough Report on Medical Education .. .. .. .. .. 17

REVIEWS OF BOOKS .. .. .. .. .. .. .. .. .. 21

EDITORIAL NOTES .. .. .. .. .. .. .. .. .. 26

OBITUARIES .. .. .. .. .. .. .. .. .. .. 27

MEDICAL LIBRARY OF THE UNIVERSITY OF BRISTOL .. .. .. .. 33

THE ONE HUNDRED AND SEVENTY-SEVENTH LIST OF BOOKS, ETC. .. 33

PUBLICATIONS RECEIVED .. .. .. .. .. .. .. .. 35

LOCAL MEDICAL NOTES—EXAMINATION RESULTS .. .. .. .. 36

420423

### The Medical Library of the University of Bristol

WITH WHICH IS INCORPORATED

**The Library of the Bristol Medico-Chirurgical Society**

---

*The following donations have been received since the publication of the list in the last issue.*

| | |
|---|---|
| Dr. Helen Aldwinckle (1) .. .. .. .. | Two volumes. |
| Sir Stanley Badock (2) .. .. .. .. | One ,, |
| British Journal of Surgery (3) .. .. .. | One ,, |
| General Medical Council (4) .. .. .. .. | Two ,, |
| Henry Phipps Institute (5) .. .. .. .. | One ,, |
| Michell Clarke Bequest (6) .. .. .. .. | Eleven ,, |
| Munro Smith Fund (7) .. .. .. .. | Two ,, |
| Royal College of Physicians (8) .. .. .. | One ,, |
| J. E. Shaw Fund (9) .. .. .. .. | Five ,, |
| University of Otago (10) .. .. .. .. | One ,, |
| John Wright & Sons Ltd. (11) .. .. .. | Two ,, |

Unbound periodicals have also been received from Dr. S. B. Adams, Captain Walter Goldfarb (U.S. Medical Corps), Professor E. W. Hey Groves, Dr. T. Milling, Professor C. Bruce Perry, Dr. J. Odery Symes, Messrs. John Wright & Sons Ltd.

### THE ONE HUNDRED AND SEVENTY-EIGHTH LIST OF BOOKS.

The figures in round brackets refer to the figures after the names of the donors. The books to which no such figures have been attached have either been bought from the Library Fund or received through the *Journal.*

| | | | |
|---|---|---|---|
| **Alyea, O. E. van** .. | *Nasal Sinuses* .. .. .. .. .. .. .. .. | (3) | 1942 |
| **Barzilai, G.** .. .. .. .. | *Atlas of Ovarian Tumours* .. .. .. .. .. | (6) | 1943 |
| **Berkeley, G. H., and Bonney, V.** | *Textbook of Gynæcological Surgery* 4th Ed. | (6) | 1942 |
| **Bernier, J. L.** .. .. .. | *A Manual for the Differential Diagnosis of Oral Lesions* .. .. .. .. .. .. .. .. .. .. .. | | 1943 |
| *Biologists in Search of Material* .. | .. .. .. .. .. .. .. .. .. .. .. .. | (1) | 1938 |
| **Bomfin, R. da C.** .. | *Tratamento das fraturas da coluna vertebral* .. | | 1942 |
| *British Pharmacopoeia* | .. .. .. .. .. .. .. .. .. .. 6th Addendum | | 1943 |
| **Burnet, J.** .. .. .. .. | *Outlines of Industrial Medicine, Legislation and Hygiene* .. .. .. .. .. .. .. .. .. .. .. | | 1943 |

*Cellona Technique* .. .. .. .. .. .. .. .. .. .. 5th Ed. 1939
**Chamberlain, E. Noble** *Symptoms and Signs in Clinical Medicine* 3rd Ed. 1943
**Chapman, H. E.** .. .. *The Law Relating to the Marketing and Sale of Medicines* .. .. .. .. .. .. .. .. .. .. 1942
*Chronic Pulmonary Disease in South Wales Coalminers.* II. Environmental Studies. (M.R.C. Special Report Series, 244.) 1943
**Clark, A. J.** .. .. .. *Applied Pharmacology* .. .. 7th Ed. (6) 1942
**Comroe, B. I., and others** *Internal Medicine in Dental Practice* 2nd Ed. 1942
**Crile, G., and Shively, F. L.** *The Hospital Care of the Surgical Patient* (11) 1943
**Curran, D., and Guttman, E.** *Psychological Medicine* .. .. .. .. .. .. 1944
*Diseases of the Basal Ganglia.* (Association for Research in Nervous and Mental Diseases) .. .. .. .. .. .. .. 1942
**Garrison, F. H., and Morton, L. R.** *A Medical Bibliography* .. .. .. .. .. 1943
**Gibbens, J. H.** .. .. *The Care of Young Babies* .. .. .. .. (6) 1943
**Gregg, A. L.** .. .. *Tropical Nursing* .. .. .. .. .. .. 2nd Ed. 1943
**Guirdham, A.** .. .. *Disease and the Social System* .. .. .. .. 1942
**Hill, A. B.** .. .. .. *Principles of Medical Statistics* .. .. 3rd Ed.
**Ilgenfritz, H. C., and Penick, R. M.** *Synopsis of the Preparation and Aftercare of Surgical Patients* .. .. .. .. .. .. 1942
*Index to the Manual of the International List of Causes of Death* (5th rev., 1938) 1944
**Jones, H. W., and Chamberlain, E. N.** *Electrocardiograms* .. .. 2nd Ed. 1943
**Langdale-Kelham, R. D., and Perkins, G.** *Amputations and Artificial Limbs* 1942
**Lundy, J. S.** .. .. *Clinical Anæsthesia* .. .. .. .. .. .. .. (6) 1943
**MacBride, W. C., and others** *Juvenile Dentistry* .. .. .. .. .. 3rd Ed. 1941
**Macgregor, A. Lee** .. *Synopsis of Surgical Anatomy* .. .. 5th Ed. 1943
**MacMurray, T. P.** .. *A Practice of Orthopædic Surgery* 2nd Ed. (6) 1944
**Maingot, R. H., and others** [Ed.] *War Wounds and Injuries* 2nd Ed. (6) 1944
**Meek, T. H.** .. .. *Guide to First-Aid Treatment of Home Guard Casualties* .. .. .. .. .. .. .. .. .. .. 1943
**Mellanby, K.** .. .. .. *Scabies* .. .. .. .. .. .. .. .. .. .. .. 1943
**Newman, G.** .. .. .. *The Building of a Nation's Health* .. .. .. 1939
**Osborne, J.** .. .. .. *Acrylic Resins in Dentistry* .. .. .. .. .. .. 1944
**Pearse, I. H., and Crocker, L. H.** *The Peckham Experiment* .. .. (1) 1944
**Pemberton, R., and Osgood, R. B.** *Medical and Orthopædic Management of Chronic Arthritis* .. .. .. .. .. .. (2) 1934
**Peters, J. P., and others** *Problems of Intestinal Obstruction* .. .. .. .. 1941
**Pickles, W. N.** .. .. *Epidemiology in a Country Practice* .. .. .. 1939
**Pohl, J. F., and Kenny, E.** *Kenny Concept of Infantile Paralysis* .. (11) 1943
**Price, D. S.** .. .. .. *Tuberculosis in Childhood* .. .. .. .. .. .. 1942
*Provisional Classification of Diseases and Injuries for use in Compiling Morbidity Statistics by the Committee on Hospital Morbidity Statics.* (M.R.C. Special Report Series, 248.) .. .. .. .. .. .. .. .. .. .. .. 1944
**Roberts, C. S. L., and others** *Sterility and Impaired Fertility* .. .. .. 1939
**Roesler, H.** .. .. .. *Clinical Roentgenology of the Cardiovascular System* 2nd Ed. 1943
**Rolleston, H. D.** [Ed.] .. *British Encyclopædia of Medical Practice* :—
*Medical Progress* .. .. (9) 1944
*Cumulative Supp.* .. .. (9) 1944
**Rolleston, H. D.** .. .. *A History of "The Practitioner," 1868-1943* .. 1943
**Rolleston, H. D., and Moncrieff, A. A.** [Eds.] *Minor Surgery* .. .. (6) 1943
*Royal College of Physicians of London—Planning Committee—Report on Medical Education* .. .. .. .. .. .. (8) 1944

| | | | |
|---|---|---|---|
| Raven, R. W. .. .. | *The Treatment of Shock* .. .. .. .. .. .. .. | | 1942 |
| Shaw, W. .. .. .. | *Textbook of Midwifery* .. .. .. .. .. .. | (6) | 1943 |
| Smart, J. .. .. .. | *Handbook for the Identification of Insects of Medical Importance* .. .. .. .. .. .. .. .. .. | | 1943 |
| Smout, C. F. V. .. .. | *The Anatomy of the Female Pelvis* .. .. | (7) | 1943 |
| Snapper, I. .. .. .. | *Medical Clinics on Bone Diseases* .. .. | (9) | 1943 |
| Solomons, B. .. .. | *A Handbook of Gynæcology* .. .. 3rd Ed. | (6) | 1934 |
| Stitt, E. R., and others | *Practical Bacteriology, Hæmatology and Animal Parasitology* .. .. .. .. .. .. 9th Ed. | | 1943 |
| *Ten Chapters on the Bath* .. .. .. .. .. .. .. .. .. .. .. .. .. .. .. .. | | | 1829 |
| Thoma, K. H. .. .. | *Traumatic Surgery of the Jaws* .. .. .. .. .. | | 1942 |
| Tidy, N. M. .. .. .. | *Massage and Remedial Exercises in Medical and Surgical Operations* .. .. .. .. 6th Ed. | | 1944 |
| Treves, F. .. .. .. | *Student's Handbook of Surgical Operations* 7th Ed. | | 1943 |
| Turner, G. Grey [Ed.] | *Modern Operative Surgery,* 3rd Ed., 2 vols. | (7) | 1943 |
| Winter, L. .. .. .. | *Operative Oral Surgery* .. .. .. 2nd Ed. | (6) | 1943 |
| Wintrobe, M. M. .. | *Clinical Hæmatology* .. .. .. .. .. .. .. .. .. | | 1942 |
| Zilboorg, G., and Henry, G. W. | *A History of Medical Psychology* .. .. | | 1941 |

### TRANSACTIONS, REPORTS, JOURNALS, ETC.

| | | |
|---|---|---|
| Annual Report of the Smithsonian Institution (for year ending 1899) .. | | 1942 |
| British Encyclopaedia of Medical Practice :— | | |
| Cumulative Supp., 1943 and 1944 .. .. | (9) | |
| Medical Progress, 1944 .. .. .. .. .. | (9) | |
| Dentists' Register, 1944 .. .. .. .. .. .. .. .. .. .. .. .. | (4) | |
| Medical Directory, 1944 .. .. .. .. .. .. .. .. .. .. .. .. .. | | |
| Medical Register, 1944 .. .. .. .. .. .. .. .. .. .. .. .. .. | (4) | |
| Proceedings of the University of Otago Medical School, Number 21, 1944 | (10) | |
| Quarterly Cumulative Index Medicus, Volume 34 (July-Dec., 1943) .. | | |
| Report (28th) of the Henry Phipps Institute, 1938-39 .. .. .. .. .. | (5) | |
| Year Book of Radiology, 1943 .. .. .. .. .. .. .. .. .. .. .. .. | | |

### Publications Received

From ASTHMA RESEARCH COUNCIL :

*Physical Exercises for Asthma.* Fourth Edition. 1943. Price 1s.

From MESSRS. HODDER AND STOUGHTON :

*Care of Tuberculosis in the Home.* By JAMES MAXWELL. Price 7s. 6d. 1943.

*Endocrine Disorders in Childhood and Adolescence.* By L. LE MERGUARD and H. TOZER. 1943. Price 15s.

From MESSRS. H. K. LEWIS & CO. LTD. :

*Common Skin Diseases.* By A. C. ROXBOROUGH, M.D. Seventh Edition. 1944. Price 18s.

*Caesarean Section.* By J. H. YOUNG. 1944. Price 6s.

*Guide to the Surgical Paper.* By R. J. MCNEILL LOVE, F.R.C.S. Second Edition. 1944. Price 6s.

From THE PRACTITIONER LTD.:

*History of "The Practitioner."* 1868–1943.

From the OXFORD UNIVERSITY PRESS:

*Notes for the R.M.O. of an Infantry Unit.* By G. P. BLACKER (Oxford War Manuals). 1944. Price 5s.

*Elementary Morphology and Physiology for Medical Students.* By J. H. WOODGER. Third Edition. 1943. Price 15s.

From MESSRS. JOHN WRIGHT & SONS LTD.:

*British Journal of Surgery.* General Index, 1934–43. Price 21s.

*British Journal of Surgery.* Numbers 123, 124. 1944. Price 12s. 6d. each.

*Physical Signs in Clinical Surgery.* By HAMILTON BAILEY, F.R.C.S. Ninth Edition. 1944. Price 25s.

### Local Medical Notes

Professor C. M. Yonge, D.Sc., has been appointed Regius Professor of Zoology in the University of Glasgow.

EXAMINATION RESULTS.

Students of the University have recently passed the following examinations:—

**University of Bristol.**—M.D.—G. H. Tovey, with Distinction.

CH.M.—Marjorie O. Dunster.

M.B., CH.B.—*First Examination:* Evelyn R. Brooks, Diana F. J. Duncan, Joan A. Higginbottom, C. D. Linton, Elizabeth Preston-Thomas, Gloria M. Rhodes, J. A. Beel, A. R. Ebanks, P. C. Farrant, Katharine, E. Fawcus, Pamela Hawkins, Pamela M. Hellings, Hilary M. K. Jenkins, G. Lucas, Ellen M. Marron, Elizabeth M. Morgan, P. A. Normandale, A. Ressel, Lucie Rosenhaugh, Margaret R. Salisbury, G. Waters, Stephanie Wright. *In Organic Chemistry:* Molly I. Govier, G. T. Salter, Naldo Sartori, H. Schnieden. *In Physics:* I. L. Brander,† D. M. Gambier.† *In Chemistry:* F. V. Eastman.† *In Biology:* Patricia M. Gilbert,† H. Schneiden.†

M.B., CH.B.—*Second Examination:* Una P. Jones, G. G. Portch. (*Section I*): G. F. Bigwood, F. G. Bolton, D. H. Bowden, W. L. Codd, D. M. Cunningham, Eudora M. R. Davies, R. S. G. Davies, W. J. Gall, N. M. Gibbs, C. E. Halliday, G. J. S. Herdman (1), Stella C. Hill, J. A. G. Holt, Catherine M. Hoyle, S. F. Iles, Mary Knowles, A. H. Laxton, Lisa E. Mandelbaum, C. A. Mills, G. E. Mitchelmore, Jean E. Morrell, J. P. Norris, E. Saphier, P. W. Seargeant (1), G. D. Teaque, Margaret J. Andrews, S. H. Dunce, J. S. R. R. Old, F. A. A. Ruggeri, S. M. Stotesbury, Sybil M. Tuffin. (*Section II*): J. H. Beatson, N. G. Glen, W. H. N. Moore, R. Simpson-White, G. Winternitz, G. F. Bigwood† F. G. Bolton,† D. H. Bowden,† W. L. Codd,† D. M. Cunningham,† Eudora M. R. Davies,† R. S. G. Davies,† W. J. Gall,† C. E. Halliday,† G. J. S. Herdman,† Stella C. Hill,† J. A. G. Holt,† Catherine M. Hoyle,† S. F. Iles,† Mary Knowles,† Lisa E. Mandelbaum,† C. A. Mills,† G. E. Mitchelmore,† Jean E. Morrell,† J. P. Norris,† E. Saphier,† P. W. Seargeant.†

M.B., CH.B.—*Final Examination* (*Section I*) *:* C. K. Bridge, Roma N. Chamberlain, W. P. Foster, Helen M. Holt, A. S. Nethercott, W. Oldham, D. V. Pleasant, M. Alms, J. B. Brierley, (Mrs.) L. M. Brierley, A. C. Edwards, E. W. Emery, (Mrs.) S. Gordon, Irene D. R. Gregory, J. R. Hawkings, D. H. Johnson, Margaret A. Lamb, G. E. P. Lee, Edith J. Lowick, J. D. Medhurst, (Mrs.) J. R. Oakes, R. A. J. Pearce, Ruth M. Rocke, Barbara F. Smith, S. A. K. Stokes, Doris G. Virgo.

M.B., CH.B.—*Final Examination :* (Mrs.) R. L. Ashley, Janet Baker, C. K. Bridge, Margaret P. Drew, J. Eskell, E. C. Hamlyn (2), Beryl M. Hinckley (2), P. A. James, R. Maggs, (Mrs.) M. Pardoe, (Mrs.) M. J. Williams, F. I. Tovey, (with Second Class Honours, Distinction in Surgery, Public Health and Forensic Medicine), R. I. Bodman, Helen M. Holt, A. S. Jones, D. M. M. Jones, M. H. Kerby, A. S. Nethercott, D. V. Pleasant, A. P. Radford, R. J. Sandry. *In Group I :* Flavia Z. L. B. James, Christine M. H. Jones, Elizabeth N. R. Wilson,† (Mrs.) J. R. Oakes, G. E. P. Lee.† *In Group II :* G. E. P. Lee, Flavia Z. L. B. James,† Christine M. H. Jones.†

B.D.S.—*First Examination :* A. J. P. Cousins.

B.D.S.—*Second Examination :* R. J. Brownlee.

B.D.S.—*Third Examination. In Dental Mechanics, etc. :* G. A. J. Comyn†, D. C. Sara†, A. F. J. Smith†, E. J. R. Morgan, P. J. Sheldrick (with Distinction in Dental Anatomy and Dental Mechanics, etc.). *Dental Anatomy only :* P. J. Blyth, W. Watson.

B.D.S.—*Final Examination. In Section I only :* H. L. Leech, I. A. Morgan (10), Evelyn M. Pitt. *In Section II* (*Dental Surgery*) *:* C. R. W. Birkett,† J. R. V. B. Gibson,† J. B. K. Mann.†

L.D.S.—*First Examination :* J. C. Fishpool, M. J. Bartlett, Margaret J. Webb.

L.D.S.—*Second Examination :* J. P. Rees.

L.D.S.—*Third Examination :* J. C. Nicholas, B. L. Evans, A. D. Baxter, L. G. D. Brown, J. P. Rees. *In Dental Mechanics, etc. :* Mary P. Hazell.†.

L.D.S.—*Final Examination*: J. Hornsby. *Section II* (*Dental Surgery*) *:* C. R. W. Birkett,† H. B. Doubt,† J. R. V. B. Gibson,† Alice B. U. Leigh,† J. B. K. Mann,† R. E. May,† R. H. Shipway,† R. C. Taylor,† J. B. Westlake,† F. E. R. Williams,† J. B. Wood,† W. E. C. Chaplin,† J. W. B. Kay,† H. L. Leech,† (Mrs.) I. Le Masurier,† I. A. Morgan,† W. H. E. Turnbull.† *Section II :* T. W. Beer, A. R. Brook, C. Gibbs, (Mrs.) I. Le Masurier, W. H. E. Turnbull, L. H. Valentine, K. W. Bowrey, Eileen Rich.

**University of Cambridge.**—M.B.—*Final Examination. Part I :* Moira K. E. Reaney, T. J. Rendle-Short, (Mrs.) H. F. Sebag-Montefiore, M. Symons. *Part II :* Moira K. E. Reaney, T. J. Rendle-Short, D. H. K. Soltau. (Pharmacology), Mari R. Simpson (née Randell), Erica W. Slade.

**Conjoint Board.**—M.R.C.S., L.R.C.P.—*First Examination :* J. A. Ardis (3), W. P. Foster (3), Helen M. Holt (3), J. A. G. Holt (4, 5), J. D. Medhurst (3), Jean R. Oakes (3), Ruth M. Rocke (3).

M.R.C.S., L.R.C.P.—*Final Examination :* J. H. E. Bergin (6, 7),† M. P. Bourke (7, 8),† E. H. Brown (6, 8, 9), Eileen M. Carey (7, 9),† Roma N. Chamberlain (6), W. R. Cole (6, 7, 8, 9),† Margot J. Copland (8, 9),† J. M. Drew (6), Margaret P. Drew (6, 8, 9), B. L. Finzel (9), Helen M. Holt (6), J. E. G. Hope-Scott (6, 7, 8, 9),† Flavia Z. L. B. James (8, 9), P. A. James (6, 9), Christine M. H. Jones (8, 9), D. M. M. Jones (6, 7, 8, 9),† P. H. Keppich (7),† R. Maggs (6), Moragh J. Noakes (6, 9), Jean R. Oakes (6), Moira K. E. Reaney (6, 7, 8), D. H. K. Soltau (6, 7, 9), M. Symons (6, 8, 9), F. I. Tovey (6, 7, 8, 9),† Marjorie J. Williams (6, 7, 8, 9),† Dorothy Willoughby (8).†

† Completing Examination.
(1) With Distinction in Anatomy.
(2) With Distinction in Public Health.
(3) Pharmacy and Materia Medica.
(4) Anatomy.
(5) Physiology.
(6) Pathology.
(7) Medicine.
(8) Surgery.
(9) Midwifery.
(10) With Distinction in Surgery.

**Out-Patients' Appointments.**—The Bristol Royal Infirmary has started a system of appointments for those referred to the Out-Patient Department (except Fracture and Orthopædic cases) for consultation. Doctors are asked to give each patient a note addressed to the member of the Staff concerned and *to tell the patient* to apply to the appointments officer for an appointment. The patient may do this by calling on the appointments officer, by writing, or by telephone (23877), between 9.0 a.m. and 5.0 p.m., or on Saturdays between 9.0 a.m. and 12.0 noon. This system does *not* apply to patients attending the General Hospital.

**Long Fox Memorial Lecture.**—The Thirty-third Long Fox Memorial Lecture was delivered by Mr. E. Watson-Williams, M.C., Ch.M., F.R.C.S., at 8.30 p.m. on Wednesday, October 18th, 1944, in the large lecture theatre of the Wills Physics Laboratory. Subject : "The English Doctor, from Smollett to Trollope."

### Bristol Medico-Chirurgical Society

### President.

C. CORFIELD, M.D. Durh.

### President-Elect.

### Committee.

*Ex-Officio* { J. A. NIXON, *C.M.G.*, B.A., M.D. Cantab., F.R.C.P. (Editor of *Journal*).

(Vacant), (Hon. Librarian).

*Elected.*

L. A. MOORE, M.B., Ch.B. Brist. (Ex-President).
R. C. CLARKE, *O.B.E.*, M.B., Ch.B.Brist.
H. H. CARLETON, M.A., M.D., B.Ch.Oxon.
A. L. TAYLOR, M.D. Lond.
P. S. MARTIN, *M.C.*
T. MILLING, M.B., Ch.B. Belf.
W. V. WOOD, *M.C.*, B.A. Cantab.
H. L. SHEPHERD, M.B., Ch.M. Brist.
W. A. JACKMAN, F.R.C.S.

### Honorary Secretary.

R. V. COOKE, Ch.M.

### Honorary Treasurer.

A. E. ILES, *O.B.E.*, M.B., B.S. Lond., F.R.C.S.

### University Library Sub-Committee.

A. R. SHORT, B.Sc., M.D. Lond., F.R.C.S.
C. BRUCE PERRY, M.D. Brist., F.R.C.P.
H. J. DREW SMYTHE, *M.C.*, M.S., M.D. Lond., F.R.C.S., F.R.C.O.G.

Medical Practitioners wishing to join the Society are requested to communicate with the Hon. Secretary, Mr. R. V. Cooke, Failand Lodge, Guthrie Road, Clifton. The Annual Subscription is £1 1s., payable to Hon. Treasurer.

---

*Extracts from Journal By-laws.*

4.—The *Journal* shall be delivered free to Subscribers and also to Members of the Society whose subscriptions are not in arrear.

6.—The Annual Subscription to the *Journal* is 9 /–, payable to Hon. Treasurer.

---

Communications intended for insertion in the *Journal* should be sent to the Editor, Dr. J. A. Nixon, 7 Lansdown Place, Clifton, Bristol.

Books for Review and Exchange Journals should be sent to the Assistant Editor, *Bristol Medico-Chirurgical Journal*, Medical Library, The University, Bristol. Reviews should be returned with the books to Mr. E. Watson-Williams, 65 Pembroke Road, Bristol 8.

Communications referring to the delivery of the *Journal* should be sent to the Hon. Treasurer, Mr. A. E. Iles, 87 Pembroke Road, Bristol 8.

Volumes of the *Journal* can be bound upon application to J. W. Arrowsmith Ltd., Quay Street, Bristol, price on request. The inclusive index for Volumes I—L may be obtained, price 3/– (postage extra).

### Bristol Medico-Chirurgical Society

### PAST PRESIDENTS

1874—75. FREDERICK BRITTAN, M.D., B.S. Dub.
1875—76. ROBERT WILLIAM COE.
1876—77. SAMUEL MARTYN, M.D. Ed.
1877—78. AUGUSTIN PRICHARD, M.D. Berlin.
1878—79. HENRY EDWARD FRIPP, M.D. Würzburg.
1879—80. WILLIAM MICHELL CLARKE.
1880—81. JOSEPH GRIFFITHS SWAYNE, M.D. Lond.
1881—82. EDWARD LONG FOX, M.D. Oxon.
1882—83. JAMES GEORGE DAVEY, M.D. St. And.
1883—84. WILLIAM JOHNSTONE FYFFE, M.D., B.A. Dub.
1884—85. GEORGE FORSTER BURDER, M.D. Aberd.
1885—86. WILLIAM HENRY SPENCER, M.D., M.A. Cantab.
1886—87. FRANCIS POOLE LANSDOWN.
1887—88. LEMUEL MATTHEWS GRIFFITHS.
1888—89. ROBERT SHINGLETON SMITH, M.D., B.Sc. Lond.
1889—90. NELSON CONGREVE DOBSON, Ch.M. Brist.
1890—91. SAMUEL HENRY SWAYNE.
1891—92. FRANCIS RICHARDSON CROSS, M.B. Lond., LL.D. Brist.
1892—93. EDWARD MARKHAM SKERRITT, M.D., B.S., B.A. Lond.
1893—94. JAMES GREIG SMITH, M.B., C.M., M.A. Aberd., F.R.S.E.
1894—95. ALFRED JAMES HARRISON, M.B. Lond.
1895—96. ARTHUR WILLIAM PRICHARD.
1896—97. ALFRED EDWARD AUST LAWRENCE, M.D., C.M. Aberd.
1897—98. JOHN EDWARD SHAW, M.B., C.M. Ed.
1898—99. ROBERT ROXBURGH, M.B. Ed.
1899—00. WILLIAM HENRY HARSANT.
1900—01. DAVID SAMUEL DAVIES, M.D. Lond.
1901—02. BARCLAY JOSIAH BARON, M.B., C.M. Ed.
1902—03. GEORGE MUNRO SMITH, M.D. Brist.
1903—04. JAMES PAUL BUSH, C.M.G., C.B.E., Ch.M. Brist.
1904—05. REGINALD EAGER, M.D. Lond.
1905—06. JOHN DACRE.
1906—07. JAMES TAYLOR.
1907—08. HENRY WALDO, M.D., C.M. Aberd.
1908—09. JOHN MICHELL CLARKE, M.D., M.A. Cantab.
1909—10. JAMES SWAIN, C.B., C.B.E., M.D., M.S. Lond.
1910—11. BERTRAM MITFORD HERON ROGERS, M.D., B.Ch., B.A. Oxon.
1911—12. CHARLES ALEXANDER MORTON.
1912—13. WALTER CARLESS SWAYNE, M.D., B.S. Lond. and Brist.
1913—14. PATRICK WATSON-WILLIAMS, M.D. Lond., M.D. (Hon. Causa) Brist.
1914—15. WILLIAM HARRY CHRISTOPHER NEWNHAM, M.A., M.B. Cantab.
1915—16. GEORGE PARKER, M.A., M.D. Cantab., LL.D. Brist.
1916—17. FRANCIS HENRY EDGEWORTH, M.A., M.D. Cantab., D.Sc. Lond.
1917—18. FREDERICK LACE.
1918—19. ROBERT GUTHRIE POOLE LANSDOWN, M.D., B.S. Durh.
1919—20. I. WALKER HALL, M.D., Ch.B. Vict.
1920—21. LEOPOLD ERNEST VALENTINE EVERY-CLAYTON, M.D. Lond.
1921—22. CYRIL HUTCHINSON WALKER, B.A., M.B. Cantab.
1922—23. JOHN ALEXANDER NIXON, C.M.G., B.A., M.D. Cantab.
1923—24. JOHN LACY FIRTH, M.D., M.S. Lond.
1924—25. JOHN ODERY SYMES, M.D. Lond.
1925—26. THOMAS CARWARDINE, M.S. Lond.
1926—27. ARTHUR LAUNCELOT FLEMMING, M.B., Ch.B. Brist.
1927—28. WALTER KENNETH WILLS, M.A., M.B., B.Ch. Cantab.
1928—29. HENRY LAWRENCE ORMEROD, M.D., B.Ch. R.U.I.
1929—30. CHARLES FERRIER WALTERS.
1930—31. DAVID CHARLES RAYNER, Ch.M. Brist.
1931—32. JOHN ROGER CHARLES, M.A., M.D. Cantab.
1932—33. ERNEST WILLIAM HEY GROVES, M.D., M.S., D.Sc., B.Sc. Lond.
1933—34. HERBERT ELWIN HARRIS, B.A., M.B. Cantab.
1934—35. A. RENDLE SHORT, B.Sc., M.D. Lond.
1935—36. ARTHUR ERNEST ILES, O.B.E., M.B., B.S. Lond.
1936—37. RICHARD CHRISTOPHER CLARKE, O.B.E, M.B. Lond.
1937—38. CLIFFORD A. MOORE, M.B., M.S. Lond.
1938—40. LEONARD AUGUSTUS MOORE, M.B., Ch.B. Brist.
1940—41. CHARLES CORFIELD, M.D. Durh., Barr.-at-Law.

1944.

### LIST OF SUBSCRIBERS

TO THE

### Bristol Medico-Chirurgical Journal.

### HONORARY MEMBER OF THE SOCIETY.

| | |
|---|---|
| Swain, James, *C.B.*, *C.B.E.*, M.D., M.S. Lond. .. .. .. | Wyndham House, Easton-in-Gordano. |

### SUBSCRIBERS.

*Those marked * are Members of the Society.*

| | |
|---|---|
| *Adams, A. W., M.S. Lond. .. .. .. .. | Elton House, Clifton, Bristol 8. |
| Adams, J. B .. .. .. .. .. .. .. .. | Kent Lodge, Eastbourne. |
| *Adams, S. B., Ph.D., M.B., Ch.B. Brist. .. | Clapton Hill, near Portishead. |
| *Aldwinckle, Helen M., M.B., Ch.B. Brist. .. | 23 Rockland Road, Downend, Bristol. |
| *Alexander, A. J. P., M.D. Belf. .. .. .. | Winsley Sanatorium, near Bath. |
| *Alexander, D. A., M.B., Ch.B. Vict. .. .. | 112 Pembroke Road, Clifton, Bristol 8. |
| Alexander, G. L., B.A., M.B., B.Ch. Cantab. | West African Medical Service, Laura, via Accra, Gold Coast. |
| *Alford, A. F., M.B., Ch.B. Brist. .. .. | Board of Education, Belgrave Square London, S.W.1. |
| Alford, H. J., M.D. Lond. .. .. .. .. | 4 Park Terrace, Taunton. |
| Ashton, L. P., M.B., Ch.B. Brist. .. .. .. | Kapsowar, P.O. Eldoret, Kenya, East Africa |
| *Aubrey, T., M.B. Lond. .. .. .. .. .. | Bitton, near Bristol. |
| *Bain, W. .. .. .. .. .. .. .. .. .. .. | 1 Greville Road, Southville, Bristol 3. |
| *Baker, Lily A., M.D., B.Ch., B.A.O., R.U.I. | 23 Pembroke Road, Clifton, Bristol 8. |
| Ball, Mrs. J. J. .. .. .. .. .. .. .. | St. Leonard's Vicarage, Redfield, Bristol 5. |
| *Barber, M. C., M.B., Ch.B. Brist. .. .. .. | Ingledene, High Street, Staple Hill, Bristol. |
| *Barbour, Lieut.-Col. R. F., R.A.M.C. .. | 13 Old Sneed Road, Stoke Bishop, Bristol. |
| *Barker, G. H., M.D., B.Sc. Lond. .. .. .. | 124 Redland Road, Bristol 6. |
| *Bates, R. M. .. .. .. .. .. .. .. .. | Purdown House, Stapleton, Bristol 5. |
| *Baxter, Ursula, M.B., Ch.B. Brist. .. .. | 167 Wells Road, Knowle, Bristol 4. |
| *Baxter, V. T., M.B., Ch.B. .. .. .. .. | 167 Wells Road, Knowle, Bristol 4. |
| *Beatson, D. H., M.B., Ch.B. Brist. .. .. | 8 Richmond Park Road, Clifton, Bristol 8. |
| *Bell, R. J. Irving .. .. .. .. .. .. .. | 5a Oakfield Road, Clifton, Bristol 8. |
| *Bergin, F. G. .. .. .. .. .. .. .. .. | 31 Oakfield Road, Clifton, Bristol 8. |
| *Berry, R. J. A., Prof., M.D., C.M. Edin. .. | 9 Derby Street, Ormskirk, Lancs. |
| *Birrell, J. A., M.D., B.S. Lond. .. .. .. | 1 Victoria Square, Bristol 8. |
| Blackett, J. F., M.D., B.S. Lond. .. .. .. | Hamsterley, Bloomfield Park, Bath. |
| Bletchly, G. Playne, M.B. Lond. .. .. | c/o Lloyds Bank, Nailsworth, Glos. |
| *Bodman, F. H., M.D., Ch.B. Brist. .. .. .. | 2 Redland Court Road, Bristol 6. |
| Bolt, R. F. .. .. .. .. .. .. .. .. .. | 70 Great Russell Street, London, W.C.1. |
| *Boulton, B. J., M.B., Ch.B. Brist. .. .. | Ham Green Hospital, Pill, near Bristol. |
| *Bown, Naomi J., M.B., Ch.B. Brist. .. .. | The Corner, Nailsea, Somerset. |
| *Bradbeer, E. G., M.B., Ch.B. Brist. .. .. | 13 Percival Road, Bristol 8. |
| Brasher, C. W. J., M.D. .. .. .. .. | Murivance, Nether Stowey, Somerset. |
| *Brinckman, Winifred P., M.B., Ch.B. Brist. | 2 Clifton Park, Bristol 8. |
| *Britton, R. B., M.B., B.S. Lond. .. .. .. | 33a Whiteladies Road, Clifton, Bristol 8. |
| *Brocklehurst, R. J., M.A., D.M. Oxon. .. | The University, Bristol 8. |
| Brown, J. E., M.B., Ch.B. Brist. .. .. .. | Coulthurst Villa, Brives Road, Maraval, Trinidad, B.W.I. |
| Bryan, C. .. .. .. .. .. .. .. .. .. | 44 Nevil Road, Bishopston, Bristol 7. |
| *Burges, R. .. .. .. .. .. .. .. .. .. | Druid's Mead, Stoke Bishop, Bristol 9. |
| *Bush, G. B., M.B., Ch.B. Brist. .. .. .. | Litfield House, Clifton, Bristol 8. |

| | |
|---|---|
| *CAIRNS, F. J. | Devon House, Whitehall Road, Bristol 5. |
| CAMERON, MARGARET, M.B., Ch.B. | 1 Dean Lane, Bristol 3. |
| *CAPPER, W. M., M.B., B.S. Lond. | Litfield House, Clifton, Bristol. |
| *CAREY, R. S., *O.B.E.*, B.A. Cantab. | 502 Bath Road, Brislington, Bristol 4. |
| *CARLETON, H. H., M.A., M.D., B.Ch. Oxon. | 1 Lansdown Road, Clifton, Bristol 8. |
| *CASSON, E., M.D., Ch.B. Brist. | Mount Pleasant, Clevedon. |
| *CHAMBERS, E. R. | 9 Clifton Park, Clifton, Bristol 8. |
| *CHARLES, J. R., M.A., M.D. Cantab. | Litfield House, Clifton Down, Bristol 8. |
| *CHITTY, H., M.S. Lond. | Naish House, Clapton-in-Gordano. |
| *CHRISTOFFERSON, P. E., M.B., Ch.B. Brist. | 14 Oakfield Road, Clifton, Bristol 8. |
| *CLAREMONT, L. E., M.D.S. Brist. | 5 Rodney Place, Clifton, Bristol 8. |
| *CLARK, Capt. DENNIS O., R.A.M.C. | Military Hospital, c/o Park Camp. Kingston, Jamaica. |
| *CLARKE, R. C., *O.B.E.*, M.B., Ch.B. Brist. | Harley Lodge, Clifton Park, Bristol 8. |
| *COATES, H. | Notts County Mental Hospital, Radcliffe-on-Trent. |
| *COCHRANE, J. H. | 11 Bryant's Hill, St. George, Bristol 5. |
| *COLLINGWOOD, F. C., M.B., Ch.B. Brist. | c/o C.M.O., British Airways, 20 College Road, Clifton 8. |
| *COOKE, ELIAZBETH M., M.D. Lond. | 53 Queen's Court, Clifton 8. |
| *COOKE, R. V., Ch.M. Brist. | Litfield House, Bristol 8. |
| *COOKSON, R. G. | Clifton College, Erdiston, Bude. |
| COOMBS, N. C. | Romaldkirk, Barnard Castle, Co. Durham. |
| *COOPER, W. R. | 13 Westfield Park, Redland, Bristol 6. |
| *CORFIELD, C., M.D. Durh. | 5 Kensington Place, Clifton, Bristol 8. |
| *CORNER, BERYL, M.D., B.S. Lond. | 3 Elton House, Rodney Place, Bristol 8. |
| *COSSHAM, W. L., M.B., Ch.B. Brist. | 3 Claremont Road, Bishopston, Bristol 7. |
| *COURTNEY, R. M., M.B., Ch.B. Glasg. | Manor House, Southmead Road, Bristol |
| *COXON, BEATRICE | 16 Richmond Hill, Clifton, Bristol 8. |
| *CRAIG, ANNE A., M.D., B.S. Lond. | 35 Queen's Court, Clifton, Bristol 8. |
| *CROOK, B. A., M.B., Ch.B. Brist. | The Laurels, Timsbury, near Bath. |
| CROOT, Lt.-Col. H. J., F.R.C.S., R.A.M.C. | Indian Gen. Hosp. (C), India Command. |
| *CROSSMAN, F. W., M.B. Durh. | White's Hill, Hambrook, near Bristol. |
| *DATTA, S. S., M.D. Brist. | Snowdon House, Fishponds, Bristol. |
| *DAVIES, E. D. D. | Standish House, Stonehouse, Glos. |
| *DAVIES, J. N. P., M.B., Ch.B. | Physiology Department, The University, Bristol. |
| *DELANEY, N., M.B., Ch.B. Manch. | The Meade, Bishopsworth, Somerset. |
| *DICKSON, W. A., M.D. St. And. | Standish House, Stonehouse, Glos. |
| *DISNEY, M., M.D. Lond. | On Active Service. |
| *DIX, C. | 14 Mortimer Road, Clifton, Bristol 8. |
| *DIXON, T. B. | R.N. Sick Quarters, Dover. |
| *DODD, GRANTHAM, M.B., B.S. | 42 Woodstock Road, Bristol 6. |
| *DUERDEN, J. R., M.B., Ch.B. Brist. | 4 Cecil Road, Bristol 8. |
| *DUTTON, HILDA R. | 405 Stapleton Road, Bristol 5. |
| *DYER, W. P. | West View, Westbury-on-Trym, Bristol. |
| *EGLINTON, J. H. C. | Street, Somerset. |
| ELLIOTT, F. P., M.B., C.M. Aberd. | 113 Grove Road, Walthamstow, London, E. 17. |
| *ELLIOTT, W. H. A., M.B., B.S. Lond. | 29 Old Sneed Avenue, Stoke Bishop, Bristol 9. |
| *EMERY, J. L. | Childrens' Hospital, Bristol 2. |
| *EVANS, G. M., M.B., Ch.B. Brist. | 117 Ashley Road, Bristol. |
| *EYRE-BROOK, A. L., M.B., M.S. Lond. | The Cottage, Portishead. |
| *FALLON, Col. J. | 35 Henleaze Gardens, Henleaze, Bristol. |
| *FELLS, G. R., M.B., Ch.B. Brist. | 19 Southmead Road, Bristol. |
| *FELLS, R. R., M.B., B.S. Lond. | 17 Mortimer Road, Clifton, Bristol 8. |
| *FENELEY, G. L., M.B., Ch.B. Brist. | Englewood, Falcondale Road, Westbury-on-Trym. |

| | |
|---|---|
| *FISHER, A. C., M.D. Brist. .. .. .. .. .. | Roan Antelope Mine, N. Rhodesia. |
| *FITZGIBBON, G. M., M.D. Lond. .. .. .. .. | 75 Pembroke Road, Clifton, Bristol 8. |
| *FORRESTER-BROWN, MAUD, M.D., M.S. Lond. | 22 Combe Park, Bath. |
| *FOSS, G. L., M.B., B.Ch. Cantab. .. .. .. | Cloud's Hill House, St. George, Bristol 5. |
| *FOX, F. E., B.A. Cantab. .. .. .. .. .. .. | Brislington House, Brislington, Bristol 4. |
| *FRASER, A. D., M.A., M.B., Ch.B. Glasg. .. | 4 Alexandra Road, Clifton, Bristol 8. |
| *FRASER, D., M.B., Ch.B. Edin. .. .. .. | Westgate Dursley, Glos. |
| *FULLER, H. L. .. .. .. .. .. .. .. .. .. | 9 Gay Street, Bath. |

| | |
|---|---|
| *GARDEN, R. R., M.A., M.B., B.Ch. Aberd. .. | 9 Harley Place, Clifton, Bristol 8. |
| GARLAND, E. B., M.B., C.M. Edin. .. .. .. | 114a Hampton Road, Redland, Bristol 6. |
| *GERRISH, NORMAN, M.B., Ch.B. Brist. .. .. | 2 Station Road, Keynsham, Som. |
| *GIBBS, J. R. .. .. .. .. .. .. .. .. .. .. | 48 Codrington Road, Bishopston, Bristol 7. |
| *GORHAM, A. P., M.B., Ch.B. Brist. .. .. .. | 53 Westbury Road, Westbury-on-Trym. |
| *GORNALL, W. A., M.D. Brist. .. .. .. .. | Ulvik, Nailsea, near Bristol. |
| *GREEN, S. B., M.B. Lond. .. .. .. .. .. .. | The Black Wicket, Westbury-on-Trym. |
| *GREENLAW, MADGE E., M.B., Ch.B. Brist. .. | 206 Redland Road, Bristol 6. |
| GRIFFITHS, CORNELIUS A. .. .. .. .. .. .. | 35 Newport Road, Cardiff. |

| | |
|---|---|
| *HAM, C. I., M.B., Ch.B.Brist. .. .. .. .. .. | 89 Stonebridge Park, Eastville, Bristol. |
| *HARRIS, H. E., M.A., M.B., B.Ch. Cantab. .. | 13 Lansdown Place, Clifton, Bristol 8. |
| *HEALES, J. N., M.B., Ch.B. Brist. .. .. .. | Holmcroft, Street, Somerset. |
| *HECTOR, F., M.D. Lond. .. .. .. .. .. .. | 1 Victoria Square, Clifton, Bristol 8. |
| *HEMPHILL, R., M.D. Dub. .. .. .. .. .. | Clonora, Blackberry Hill, Fishponds. |
| *HENDERSON, JAMES, M.B., Ch.B. Aberd. .. | The City Sanatorium, Yardley Road, Birmingham. |
| *HERAPATH, C. E. K., *M.C.*, M.D.Lond. .. | Ormlie, Clifton Down, Bristol 8. |
| *HEWER, Professor T. F., M.D. Brist. .. .. | Canynge Hall, Bristol 8. |
| *HILL, WINIFRED, M.B., Ch.B. Brist. .. .. | c/o Lloyd's Bank, Ilford. |
| *HOFFMAN, H. LOVELL, B.A., M.B., B.Ch. Cantab. | R.N. Hospital, Invergordon. |
| *HOLLAND, W. A. L. .. .. .. .. .. .. .. .. | 1 Upper Belgrave Road. Clifton. |
| *HOOKER, J. A., Colonel, M.B., Ch.B.Brist. .. | 16 St. Edyth's Road, Sea Mills 9. |
| *HOSKEN, J. G. F... .. .. .. .. .. .. .. | Uley, Glos. |
| HOSPITAL, BRISTOL GENERAL (*Secretary*, T. W. GREGG). | |
| *HOUGHTON, LYDIA M., M.B., B.S. Lond. .. | 74 Church Road, Redfield, Bristol 5. |
| *HOWELL, T. E. .. .. .. .. .. .. .. .. .. | 6 Richmond Hill, Bristol 8. |
| *HUGHES, F. W. TERRELL, M.B., Ch.B. Brist. | Chew Magna, Somerset. |
| *HUGHES, MARGUERITE G., M.B., Ch.B. Brist. | Walnut Tree Farm, East Dundry, Som. |
| *HUNTER, F. S. .. .. .. .. .. .. .. .. .. | c/o Messrs. J. Wright & Sons Ltd., Publishers, 28 Orchard Street, Bristol 1. |
| *HYATT, ANNIE W., M.B., B.S. Lond. .. .. | Merrymead, Shepton Mallet, Som. |

| | |
|---|---|
| *ILES, ANNA, M.B., Ch.B. Brist. .. .. .. | 87 Pembroke Road, Clifton, Bristol 8. |
| *ILES, A. E., *OB.E.*, M.B., B.S. Lond. .. .. | 87 Pembroke Road, Clifton, Bristol 8. |
| INFIRMARY, BRISTOL ROYAL (*Secretary*, E. C. SMITH). | |
| INGLE, C. DURBAN .. .. .. .. .. .. .. | Somerton, Somerset. |
| *ISSERLIN, B. .. .. .. .. .. .. .. .. .. .. | 2 College Road, Bristol 8. |

| | |
|---|---|
| *JACKMAN, W. A., *T.D.*, M.B., Ch.B. Brist. .. | 15 Mortimer Road, Clifton, Bristol 8. |
| *JAMES, APRIL D., M.B., Ch.B. Brist. .. .. | (Address unknown.) |
| *JAMES, J. A., M.D. Lond. .. .. .. .. .. .. | Litfield House, Clifton Down. Bristol 8. |
| *JAMES, J. A., Senr. .. .. .. .. .. .. .. | Dusk, Elmlea Avenue, Stoke Bishop, Bristol, 9. |
| *JENKINS, F. G., M.B., Ch.B. Brist. .. .. .. | 51 Redcliff Hill, Bristol 1. |
| *JENKINS, R. D. .. .. .. .. .. .. .. .. .. | The Copper Beeches, Almondsbury, near Bristol. |
| *JENKINS, P. K., M.B., Ch.B. .. .. .. .. .. | R.A.M.C. |

| | |
|---|---|
| *JOHNSON, R. G., M.B. Lond. | 119 Redland Road, Bristol 6. |
| *JOLL, C. A., M.D., M.S. Lond. | 64 Harley Street, London, W.1. |
| *JONES, MEGAN E. | Royal Infirmary, Bristol 2. |
| *KEMM, N. F., M.B., B.S. Lond. | 200 Redland Road, Durdham Park, Bristol, 6 |
| *KERSLEY, G. D., M.D. | 6 The Circus, Bath. |
| KING, E. F. | 2 Upper Wimpole Street, London, W.1. |
| *KINGSTON, S. H., M.B., Ch.B. Brist. | 3 Whiteladies Road, Clifton, Bristol 8. |
| *KYLE, H. G., M.A., M.D., B.Ch. Oxon. | 31 Westbury Road, Bristol. |
| *LACE, F | 5 Gay Street, Bath. |
| LALONDE, T. P., M.B., Ch.B. Brist. | Wykeham House, Romsey, Hants. |
| *LEES, E. LEONARD, M.D., C.M. Edin. | 28 Downs Park West, Bristol 6. |
| *LEGAT, R. EDDOWES, M.B., C.M. Edin. | Hampden, St. Briavels, Glos. |
| *LEWIS, F. J., M.B., Ch.B. Brist. | Canynge Hall, Bristol 8. |
| *LINTON, MARION S., M.B. Lond. | 1 Brecon Road, Henleaze, Bristol. |
| *LOGAN, H. B. | Elmwood, Aberdeen Road, Cotham Bristol 6. |
| *LOXTON, S. D., M.B., Ch.B. | |
| *LUCAS, J. E. | c/o 132 York Road, Bristol 3. |
| *LUCAS, J. J. S., M.D. Lond. | 186 Wells Road, Knowle, Bristol 4. |
| *LUCAS, J. V. | 186 Wells Road, Knowle, Bristol 4. |
| *LUCAS R. P., M.B., Ch.B. Brist. | Northways, Fallodon Way, Henleaze. |
| *MCKENZIE, W. G., *M.C.* | 11 Bath Road, Reading. |
| *MACLACHLAN, A. M., M.B., Ch.B. Glasg. | 65 Redcliff Hill, Bristol 1. |
| *MARSHALL, A. H., M.B., Ch.B. Brist. | 281 Canford Lane, Bristol. |
| *MARWOOD, S. F., M.D., B.S. | Troy House, Kingswood, Bristol. |
| *MCLANNAHAN, J. G. | The Mount, Stonehouse. |
| *MACLAREN, CATHERINE J., M.B., Ch.B. Edin. | Hereford House, Clifton Down. |
| MACLEAN, SIR EWEN J., M.D., C.M. Edin. | 12 Park Place, Cardiff |
| *MACLEOD, G., M.A. Glas., M.D., M.B. | Wargrave, Clevedon, Somerset. |
| *MARSHALL, A. H., M.B., Ch.B. Brist. | 281, Canford Lane, Bristol. |
| MARSHALL, A. L., M.B., B.A. Cantab. | Laxfield, Woodbridge, Suffolk. |
| *MARTIN, P. S., *M.C.* | Victoria Quadrant, Weston-super-Mare. |
| MASON, J. JOHNSON | Brentry House, Brentry. |
| *MATHESON, N. R., M.B., Ch.B. | 37 Abbey Road, Westbury-on-Trym. |
| MIALL, C. L'O | Elm Hayes, Paulton, near Bristol. |
| *MILLING, T., M.B., Ch.B. Belf. | 1 Vicarage Road, Southville, Bristol 3. |
| *MOORE, C. A., M.B., M.S. Lond. | South Lodge, Kingsweston Road, Henbury. Bristol. |
| *MOORE, L. A., M.B., Ch.B. Brist. | Hillsborough, Cotham Park, Bristol 6. |
| *MOORE, R. H., M.B., Ch.B. Brist. | White Cross Court, Wells Road, Bristol 4. |
| *MUNDY, G. S., M.B., B.Ch. Brist. | Homewood, Coombe Dingle, Bristol. |
| *MYLES, Q. St. L., M.A., B.M., B.Ch. Oxon. | 7 Apsley Road, Clifton, Bristol 8. |
| *NICHOLSON-LAILEY, J. R. | Elmfield House, South Road, Taunton. |
| *NIXON, DOREEN, M.B., B.S. Lond. | 7 Lansdown Place, Clifton, Bristol 8. |
| *NIXON, J. A., *C.M.G.*, M.D. Cantab. | 7 Lansdown Place, Clifton, Bristol 8. |
| *NOBLE, J. I., M.B., Ch.B. Liverp. | 102 Pembroke Road, Clifton, Bristol 8. |
| *NORMAN, R. M., M.D. Brist. | Highwood, Stapleton Park, Bristol. |
| *O'DONOHOE, MONICA A., M.B., Ch.B. | 1 Beaconsfield Road, Bristol 8. |
| *ORMEROD, G. L. M.A., M.B., B.Ch. Cantab | 2 Henleaze Road, Westbury-on-Trym, Bristol. |
| *ORR-EWING, H. J., *M.C.*, M.D. Lond. | 1 Beaufort Buildings, Clifton, Bristol 8. |
| *PALIN, A. G., B.M., B.Ch. Oxon. | Litfield House, Clifton Down, Bristol 8. |
| *PARKES, J. A., M.B., Ch.B. Liverp. | 8 South Road, Redland, Bristol 6. |

| | |
|---|---|
| *PARKES, P. W. J. .. .. .. .. .. .. .. | 164 Filton Avenue, Bristol 7. |
| PARKINSON, Lt.-Col. G. S., *D.S.O.*, R.A.M.C. | London School of Hygiene and Tropical Medicine, Keppel Street, London, W.C. 1. |
| *PARRY, R. H., M.B., B.S.Lond. .. .. .. | Public Health Offices, Bristol 1. |
| PARSONS, Sir J. H., M.B., B.S., B.Sc. Lond... | 54 Queen Anne Street, London, W. |
| *PAUL, R. G. .. .. .. .. .. .. .. .. .. | 23 Pembroke Road, Bristol 8. |
| *PAULI, C. H. .. .. .. .. .. .. .. .. .. | Pagan's Hill Farm, Chew Stoke. |
| *PERRY, B., M.D., Ch.B.Brist. .. .. .. .. | Canynge Hall, Clifton, Bristol 8. |
| PHELPS, P. C., M.B., Ch.B. .. .. .. .. | 23 St. Alban's Road, Bristol 6. |
| *PHILLIPS, P., M.B., Ch.B. Brist. .. .. .. | Southmead Hospital, Bristol. |
| *PIRRIE, A. L., M.B., Ch.B. Glasg. .. .. | 528 Kensington Hill, Bristol. |
| *POCOCK, J. A., M.A., M.B., B.Ch. Cantab. .. | 75 Drayton Gardens, London, S.W. 10. |
| PORTER, C. P. .. .. .. .. .. .. .. .. .. | 28 Mill Street, Kidderminster. |
| *POTTER, MABEL F., M.B., Ch.B. Brist. .. .. | 17 Royal Park, Clifton, Bristol 8. |
| *POWELL, H. F., M.D. Brux. .. .. .. .. .. | Ellenborough Park, Weston-super-Mare. |
| *PRICE, C. H. G., M.B., Ch.B. Brist. .. .. | Claverham House, Claverham, Yatton. |
| *PRICE, N. L., M.B., Ch.B. Brist. .. .. .. | Litfield House, Clifton Down, Bristol 8. |
| *PRIDIE, K. H., M.B., B.S. Lond. .. .. .. | 58 St. John's Road, Clifton, Bristol 8. |
| *PROWSE, D. C., M.B., Ch.B. Brist. .. .. .. | Wigmore House, Thornbury, near Bristol. |
| | |
| *RANKIN, P. C., M.B., Ch.B. Glasg. .. .. .. | Lawrence Hill House, Bristol. |
| *RAYNER, D. C., Ch.M. Brist. .. .. .. .. | 9 Lansdown Place, Clifton, Bristol 8. |
| *ROBERTS, J. A. F., M.A. Cantab., M.B., Ch.B., D.Sc. Edin. | Stoke Park Colony, Bristol. |
| *ROBERTSON, D., M.B., Ch.B. Edin. .. .. .. | 2 Knowle Road, Knowle, Bristol 4. |
| *ROBERTSON, L. G., M.B., Ch.B. .. .. .. | 162 Redland Road, Bristol 6. |
| *ROGERS, H., M.D. Brist. .. .. .. .. .. .. | 91 Old Park Ridings, Grange Park, N.21. |
| *ROLFE, R., B.A., M.B., B.C. Cantab. .. .. | Park House, St. Andrew's Road, Avonmouth. |
| *ROWLANDS, R. D. .. .. .. .. .. .. .. | Connaught Military Hospital, Knaphill, Surrey. |
| *ROWLEY, A. T. .. .. .. .. .. .. .. .. | 13 Walliscote Road, Weston-super-Mare. |
| *RUDOLF, MAJOR G. DE M. .. .. .. .. .. | R.A.M.C. |
| *RYAN, P. B., M.B., Ch.B. .. .. .. .. .. | The Paddock, Frenchay. |
| | |
| *SAMPSON, O. E. L., M.B., Ch.B. Brist. .. .. | 77 Stackpool Road, Bristol 3. |
| *SCARFF, G., M.B., Ch.B. Edin. .. .. .. .. | 83 Pembroke Road, Clifton, Bristol 8. |
| *SHEPHERD, H. L., M.B., Ch.M. Brist. .. .. | 79 Pembroke Road, Clifton, Bristol 8. |
| *SHORT, A. R., F.R.C.S., M.D. Lond. .. .. .. | 69 Pembroke Road, Clifton, Bristol 8. |
| *SMITH, T. W., M.D. Lond. .. .. .. .. .. .. | 6 Oakley, Claverton Down, Bath. |
| *SMYTHE, H. J. D., *M.C.*, M.D., M.S Lond. | On Active Service. |
| *SNAWDON, J. W. E., M.B., Ch.B., B.D.S. .. .. | Litfield House, Clifton, Bristol 8. |
| *SOLTAU, H. K. V., M.D. Lond. .. .. .. | 19 The Avenue, Clifton, Bristol 8. |
| *SOMERS, MARY A. E. .. .. .. .. .. .. .. | 2 Ashcombe Road, Weston-super-Mare. |
| *SPOOR, A., M.B., B.Ch. Cantab. .. .. .. | The Hydro, Bristol 1. |
| *STEPHEN, R. J. .. .. .. .. .. .. .. .. | 92 Redland Road, Bristol 6. |
| *STROVER, H. W. M., *O.B.E.*, M.B., Ch.B. Aberd. | 90 Redland Road, Bristol 6. |
| *STRUTHERS, A. J., M.B., Ch.B. Glasg. .. .. | 100 Church Road, Redfield, Bristol 5. |
| *STRUTHERS, GEO., M.B., Ch.B. Glasgow .. .. | 100 Church Road, Bristol 5. |
| *SUTTON, G. E. F., M.D. Lond. .. .. .. .. | 1 Pembroke Road, Clifton, Bristol 8. |
| *SYMES, J. O., M.D. Lond. .. .. .. .. .. | 6 Pembroke Vale, Clifton, Bristol 8. |
| | |
| *TALLACK, MAJOR R. J. K., R.A.M.C. .. .. | (Kenya) Field Amb., A.P.O., E.A. Command. |
| *TASKER, D. G. C., M.B., M.S. Lond. .. .. | 9 College Fields, Clifton, Bristol 8. |
| *TAYLOR, A. L., M.D. Lond. .. .. .. .. | The General Hospital, Bristol 1. |
| TAYLOR, A. L. .. .. .. .. .. .. .. .. | The Royal Infirmary Bristol 2. |
| THIN, J. .. .. .. .. .. .. .. .. .. .. | 54 and 55 South Bridge, Edinburgh. |
| *TODD, A. T., *O.B.E.*, M.D. Edin. .. .. .. | Royal Park House, Clifton, Bristol 8. |
| TRINICK, RICHARD H. .. .. .. .. .. .. | 42 Cherry Valley Gardens, Belfast. |
| TRIPP, DOROTHY M. H. .. .. .. .. .. | Mission Hospital, Karim Nagar, Nizam's Dominions, South India. |
| *TRYON, VICTORIA S., M.B., Ch.B. Brist. .. | 3 Harley Place, Clifton, Bristol 8 |

| | |
|---|---|
| *WAGSTAFFE, E. M., M.B., Ch.B. | Winford Orthopædic Hospital. |
| *WANSBROUGH, T. B., M.B., Ch.B. Brist. | 23 Pembroke Road, Clifton, Bristol 8. |
| *WALKER, CYRIL H., B.A., M.B. Cantab. | Harewood, Clapton-in-Gordano, Som. |
| *WALTERS, C. F. | Downover, Walton Down, near Clevedon. |
| WAKE, S. C., M.B., Ch.B. Brist. | "Stanhope," Bideford, N. Devon. |
| *WARD, H. W. | Chipping Manor, Wotton-under-Edge. |
| *WARREN, R., M.A., M.D. Oxon. | Heathgates, Weston-super-Mare. |
| *WATSON-WILLIAMS, E., *M.C.*, B.A., M.B. Cantab., Ch.M. Brist. | 65 Pembroke Road, Clifton, Bristol 8. |
| WATTS, J. B. V. | Frantzina's Rust, Barberton, Transvaal, S. Africa. |
| WEDDELL, C. W. | Boydville, 46 Miller Street, West Melbourne, Australia. |
| *WIGODER, SYLVIA, M.A., M.D., Ph.D. | General Hospital, Bristol 1. |
| *WILBOND, R. G. | 76 Chesterfield Road, St. Andrew's Pk., Bristol. |
| *WILLIAMS, I., M.B., Ch.B. Brist. | "Ardrem," Hill View, Henleaze, Bristol. |
| *WILLIAMS, S. R., M.B. Lond. | Buckfastleigh, Devon. |
| *WILLWAY, F. W., M.D., M.S. Lond. | Bristol Royal Infirmary. |
| *WOOD, D. | 105 Pembroke Road, Clifton, Bristol 8. |
| *WOOD, URSULA, M.B., Ch.B. | Henley Lodge, Yatton, Som. |
| *WOOD, W. V., *M.C.*, B.A. Cantab. | Henley Lodge, Yatton, Som. |
| *WOODMAN, DOROTHY, M.D. Lond. | 10 Tyndall's Park Road, Clifton, Bristol 8. |
| *WOOLLEY, W., M.B., Ch.B. Brist. | 239 Cranbrook Road, Redland. |
| *WRIGHT, A. J. M., M.B., B.S. Lond. | Harley Cottage, Clifton Park, Clifton, Bristol 8. |
| *WRIGHT, WINIFRED M., M.B., B.S. Lond. | Merrymead, Shepton Mallet, Som. |
| *ZEALAND, L., M.D. Man. | Brecon Lodge, Westbury-on-Trym, Bristol. |

### *HONORARY VISITING MEMBERS.*

BRIDGES, MAJOR ARTHUR BRODIE HAMILTON, R.A.M.C., *O.B.E.*

COLSON, SURGEON REAR-ADMIRAL HENRY ST. CLAIR, *O.B.E.*, M.D. Lond.

WHITBY, COLONEL LIONEL ERNEST HOWARD, *A.M.S.*, *C.V.O.*, *M.C.*, M.D. Cantab.

WORTHINGTON, COLONEL FRANK, *A.M.S.*, *D.S.O.*, *O.B.E.*, M.B., B.Ch. Cantab.

And their staffs.

### Index to Vol. LXI.

Blind typist, which can only be done by, 26

Bristol University.—
Examination Results, 36
Medical Library, 33–36

English Doctors from Smollett to Trollope. — E. Watson - Williams (Long Fox Memorial Lecture), 38

Goodenough Report on Medical Education, 17

Grants for Research, 26

Long Fox Memorial Lecture.—
(32nd).—C. Bruce Perry, 1
(33rd).—E. Watson-Williams, 38

" Look and See,"—E. Watson-Williams, 11

Medical Education, Goodenough Report on, 17

Obituaries.—
Edward Mills Grace, 27
E. W. Hey Groves, 32
Leslie Roland Jordan, 27
Frederic Percival Mackie, 28
Frederick John Poynton, 29
Francis Wilfred Willway, 30

Out-patients Departments, 38

Perry, C. Bruce.—32nd Long Fox Memorial Lecture, 1

Professors.—
Dr. J. E. Harris, 26
Dr. Wilson Baker, 26

Reviews of Books, 21

Social Aspects of Acute Rheumatism.—C. Bruce Perry (32nd Long Fox Memorial Lecture), 1

Tyson, Edward, 1650–1708, 15

Watson-Williams, E.—
33rd Long Fox Memorial Lecture, 38
" Look and See," 11

